# Brazilian PENSA protocol: practical guide to difficult communication

**DOI:** 10.1590/1806-9282.20240674

**Published:** 2024-09-16

**Authors:** Henrique Gandara Canosa

**Affiliations:** 1 Instituto do Câncer do Estado de São Paulo – São Paulo (SP), Brazil.

## INTRODUCTION

Patients need to be properly guided by the healthcare team when facing medical diagnoses. They must understand all aspects in detail, ranging from the meaning of the disease and the impact of this diagnosis on their lives and those of their families to how to make decisions from then on. The multidisciplinary medical team must communicate assertively so that patients understand everything that involves their treatment and prognosis.

Communication techniques are not always well taught and studied during undergraduate courses, and healthcare professionals are often poorly prepared to communicate directly and effectively. On some occasions, these professionals do not have the proper preparation to conduct difficult conversations, such as, for example, communicating bad news. Several empirical studies have shown that doctor–patient communication is inferior to what is considered adequate, and two of the main causes are lack of experience and reluctance to deal with patients’ feelings^1-3^. Good communication reduces stress and increases the satisfaction of healthcare service users^4^.

Regarding bad news, there are basically three types in medicine: unfavorable diagnosis, unfavorable prognosis, and death of the patient. Each contains a message that will negatively affect the lives of those who receive it^5^. Not only receiving but also communicating news, especially the bad ones, generates a certain type of discomfort, which is why numerous communication tools have been created over the years to minimize tension and improve communication efficiency^5,6^. The most commonly used communication protocols are SPIKES^7^ (S—Setting, P—Patient's perception, I—Invitation, K—Knowledge, E—Exploring/Empathy, S—Strategy/Summary), BREAKS^1^ (B—Background, R—Rapport, E—Explore, A—Announce, K—Kindling and S—Summarize), and ABCDE^8^ (A—Avoid Shaming/Personal Opinions, B—Build a Rapport, C—Choose a Communication Approach, D—Develop a Debriefing Content, E—Ensure the Ergonomics of Debriefing), but many are complex and difficult to apply in clinical practice.

For this reason, the author developed a Brazilian protocol that is easy to memorize and use, which aims to facilitate communication between healthcare professionals and patients/relatives: PENSA (P—Preparação; E—Escuta empática; N—Notícia difícil; S—Síntese; A—Atitude). This protocol is also easily adaptable to English: P—preparation; E—empathetic listening; N—news; S—summary; A—action.

## PENSA PROTOCOL

PENSA is an acronym created by the author to make it easier to memorize the steps to follow for effective communication, which will be explained below. A schematic for the practical application of the PENSA protocol can be found in detail in Table 1 and summarized in Table 2.

**Table 1:** PENSA protocol.

Preparation	Preparation of the environment: Suitable place with chairs, water, and tissue paper Preparation of professionals: Understand the case Get to know the patient Get prepared emotionally and physically Turn off your phone Dress appropriately Align with the team on the objective of the meeting Get to know the patient's social/family situation Introduce yourself to the patient and family members Patient and family preparation: Schedule the meeting in advance Briefly explain the objective of the meeting Give family members time and space to introduce themselves
Empathetic listening	Non-verbal communication: Use body mirroring technique (mirroring the position of arms, legs, facial expressions) Keep your arms uncrossed Maintain eye contact (at least 50% of the conversation time) Raise eyebrows at first eye contact Keep the neck visible Move your head in an affirmative way Tone of voice: Mirror the tone of voice of the person you are talking to, giving preference to a calm and low tone of voice Use open-ended questions, to know: The evolution of the disease The treatments performed The patient's decline or improvement The family/patient's understanding Use pauses during conversation (moment of silence) Use expressions of understanding, such as: "I understand" "Uh-huh" "Yes, I understand" "Good, I understand" "OK, understood"
News	After a piece of news, take a break (silence) – the family/patient should be the one to break the silence Use simple words and expressions Use affirmative expressions: "We can treat it this way" "There's treatment X that can be performed" "We can use this medication to control this symptom" Clarify concerns and questions
Summary	Summarize everything that was said during the meeting Assess if there are any questions about the topic covered
Action	Explain to the family/patient what actions will be taken from that meeting Say goodbye and thank everyone for their participation Write down the meeting in the patient's medical record

**Table 2:** PENSA Protocol – overview.

Preparation	Of the environment Of the professionals Of the patient and the family
Empathetic listening	Non-verbal communication Tone of voice Open-ended questions Pauses/silence Confirm that everyone understands
News	Simple words and expressions Pause/silence after the news Affirmative expressions Clarify doubts
Summary	Rundown Assess if they understand Clarify doubts
Action	Actions that will be taken after the meeting Farewell and thank the family Make notes in the medical record

### P—Preparation

Before interacting with the patient and their family, it is necessary to prepare the environment, whatever it may be: a reserved room in the hospital, a doctor's office, the hospital room in which the patient is hospitalized, or even the patient's home. The place must have chairs or sofas so that everyone can sit comfortably for the conversation, as well as water and tissue paper.

The preparation of professionals is extremely important. It is necessary to have a previous alignment meeting between all professionals, so they share the same objectives and understanding of the case. It is also crucial they get to know the patient's case and their social and family situation in detail. Emotional preparation is another fundamental piece to be able to conduct the conversation in the best possible way, knowing that the patient and their family members may be emotionally weak due to the health situation. Cell phones should be turned off to minimize any external interference. All members of the staff should introduce themselves to the patient and family members when they arrive at the meeting place.

The preparation of the family is also part of successful communication. The meeting must be scheduled previously, and everyone must know its content and objectives. The family and the patient must be instructed to prepare questions for the team, including writing them down so that they can ask all the questions during the conversation.

The main steps of the **Preparation** stage are as follows:

- Preparation of the environment:
- Suitable place with chairs, water, and tissue paper.
- Preparation of professionals:
- Understand the case;
- Get to know the patient;
- Get prepared emotionally and physically;
- Turn off your phone;
- Dress appropriately;
- Align with the team on the objective of the meeting;
- Get to know the patient's social/family situation;
- Introduce yourself to the patient and family members.
- Patient and family preparation:
- Schedule the meeting in advance;
- Briefly explain the objective of the meeting;
- Give family members time and space to introduce themselves.

### E—Empathetic listening

During the empathetic listening stage, some essential items for successful communication are observed. Communication is composed of 55% body language, 38% tone of voice, and only 7% the words themselves according to studies by Professor Albert Mehrabian^9^. Therefore, attention should be paid to the body language at the meeting with the patient and their family members. The author considers some attitudes crucial for the harmony between the team, the patient, and their relatives. These attitudes include keeping arms uncrossed, indicating a position of tranquility and comfort, maintaining eye contact for at least 50% of the conversation time, raising eyebrows at first eye contact, and keeping the neck visible so that the other party instinctively does not see the team as a threat, making affirmative movements with your head to show that you are following the conversation, and using the body mirroring technique (mirroring the position of the arms, legs, and facial expressions).

As mentioned above, the tone of voice accounts for 38% of communication. Usually, to communicate more effectively, mirror the tone of voice by adjusting the speech speed and volume based on who you are speaking to. Ideally, in hospitals, you should always keep the tone of voice calm and volume low. Avoid interrupting people's speech as much as possible since the goal is to understand their queries and anxieties and provide the best help. This is a very important step: Allowing patients and their families to express their emotions is essential for the team to offer the necessary support.

The main steps of the **Empathetic listening** stage are as follows:

- Non-verbal communication:
- Use body mirroring technique (mirroring the position of arms, legs, facial expressions);
- Keep your arms uncrossed;
- Maintain eye contact (at least 50% of the conversation time);
- Raise eyebrows at first eye contact;
- Keep the neck visible;
- Move your head in an affirmative way.
- Tone of voice:
- Mirror the tone of voice of the person you are talking to, giving preference to a calm and low tone of voice.
- Use open-ended questions, to know:
- The evolution of the disease;
- The treatments performed;
- The patient's decline or improvement;
- The family/patient's understanding.
- Use pauses during conversation (moment of silence).
- Use expressions of understanding, such as:
- "I understand."
- "Uh-huh."
- "Yes, I understand."
- "Good, I understand."
- "OK, understood."

## N—News

Communicating bad news is often a difficult task. It is important to ensure that patients and their families have realistic expectations. For this reason, the previous stage of active listening is extremely important, as it is at that moment that the medical team can gain a clear understanding of the patient's understanding of their disease, prognosis, and the possibility of curative treatment.

When breaking the news, even when the patient already suspects what is happening, the team must remain silent for a while, so they absorb what has been communicated.

Using simple words and expressions is key since most people do not understand medical terms. It is also important to use affirmative expressions and finally clarify their concerns.

The main steps of the **News** stage are as follows:

- After a piece of news, take a break (silence)—the family/patient should be the one to break the silence.
- Use simple words and expressions.
- Use affirmative expressions:
- "We can treat it this way."
- "There's treatment X that can be performed."
- "We can use this medication to control this symptom."
- Clarify concerns and questions.

## S—Summary

At this point, the conversation is already ending. A summary of everything said should be provided for a shared understanding and comprehension of the next steps. Before the meeting ends, everyone must ensure no questions are left.

The main steps of the **Summary** stage are as follows:

- Summarize everything that was said during the meeting.
- Assess if there are any questions about the topic covered.

## A—Action

To conclude, it is necessary to explain the actions that will be taken after that meeting to the patient and their family, say goodbye, and thank everyone for their participation. The communication content should be registered in the patient's medical record, as well as the time to indicate when the meeting occurred and, from that moment, the team should initiate any necessary follow-up steps.

The main steps of the **Action** stage are as follows:

- Explain to the family/patient what actions will be taken from that meeting.
- Say goodbye and thank everyone for their participation.
- Write down the meeting in the patient's medical record.

## FINAL CONSIDERATIONS

Healthcare teams must be aligned with each other and know exactly what and when to communicate. For this process to be successful, they must understand the environment and the people who will receive the news as accurately as possible to reality.

Bad news can cause strong emotions in patients and their families, affecting their perceptions of information, but the proper management of this communication can help these people adapt to the situation and face the next stages of care.
